# Specialized pro-resolving mediators and cancer: news and promises from the other side of the moon

**DOI:** 10.3389/fphar.2026.1907377

**Published:** 2026-10-01

**Authors:** V. Gatti, G. Colella, F. Violante, V. M. Fazio, M. Cioce

**Affiliations:** 1 Department of Medicine, Laboratory of Molecular Medicine and Biotechnology, Campus Bio Medico University of Rome, Rome, Italy; 2 Institute of Translational Pharmacology, National Research Council of Italy (CNR), Rome, Italy

**Keywords:** cancer, efferocytosis, resolution of inflammation, specialized pro-resolving mediators, SPM, therapy resistance, tumor microenvironment

## Abstract

In physiological settings, inflammation is not passively terminated but actively resolved through coordinated endogenous programs. A central component of this response is the biosynthesis of specialized pro-resolving mediators (SPMs), including lipoxins, resolvins, protectins, and maresins. These SPMs arise from polyunsaturated fatty acid precursors that are partially shared with those of classical pro-inflammatory mediators, such as prostaglandins and leukotrienes. Rather than broadly suppressing immunity, SPMs promote the restoration of tissue homeostasis by limiting excessive leukocyte recruitment, enhancing efferocytosis, and reprogramming inflammatory responses. Inflammation is a recognized enabler of cancer, contributing to the main hallmarks of tumor progression, such as sustained proliferative signaling, angiogenesis, and immune evasion. A chronically inflamed tumor microenvironment fosters cancer progression in a complex, tumor- and stage-specific manner. This persistent inflammatory context has supported the prescient view of tumors as “wounds that do not heal.” While the pro-tumorigenic functions of inflammatory lipid mediators, such as prostaglandin E_2_, are well established, the contribution of defective resolution programs to cancer progression remains less clear. This review examines the lipid mediator networks linking the initiation and resolution of inflammation in solid tumors, focusing on the mechanistic roles of SPMs in the tumor microenvironment and the emerging translational opportunities to restore pro-resolving signaling. This supports a re-evaluation of cancer-associated inflammation from the perspective of failed resolution, opening avenues for therapeutic strategies targeting resolution to mitigate therapy resistance. We develop this idea by bringing together direct and indirect scientific evidence, explicitly distinguished throughout the text and summarized in a dedicated evidence table, and by focusing on a prospective pharmacological scenario. This is a narrative review; its scope, literature search strategy, and limitations are described in Sections 1.1, 1.2, and 8.

## Graphical Abstract

**Figure:**
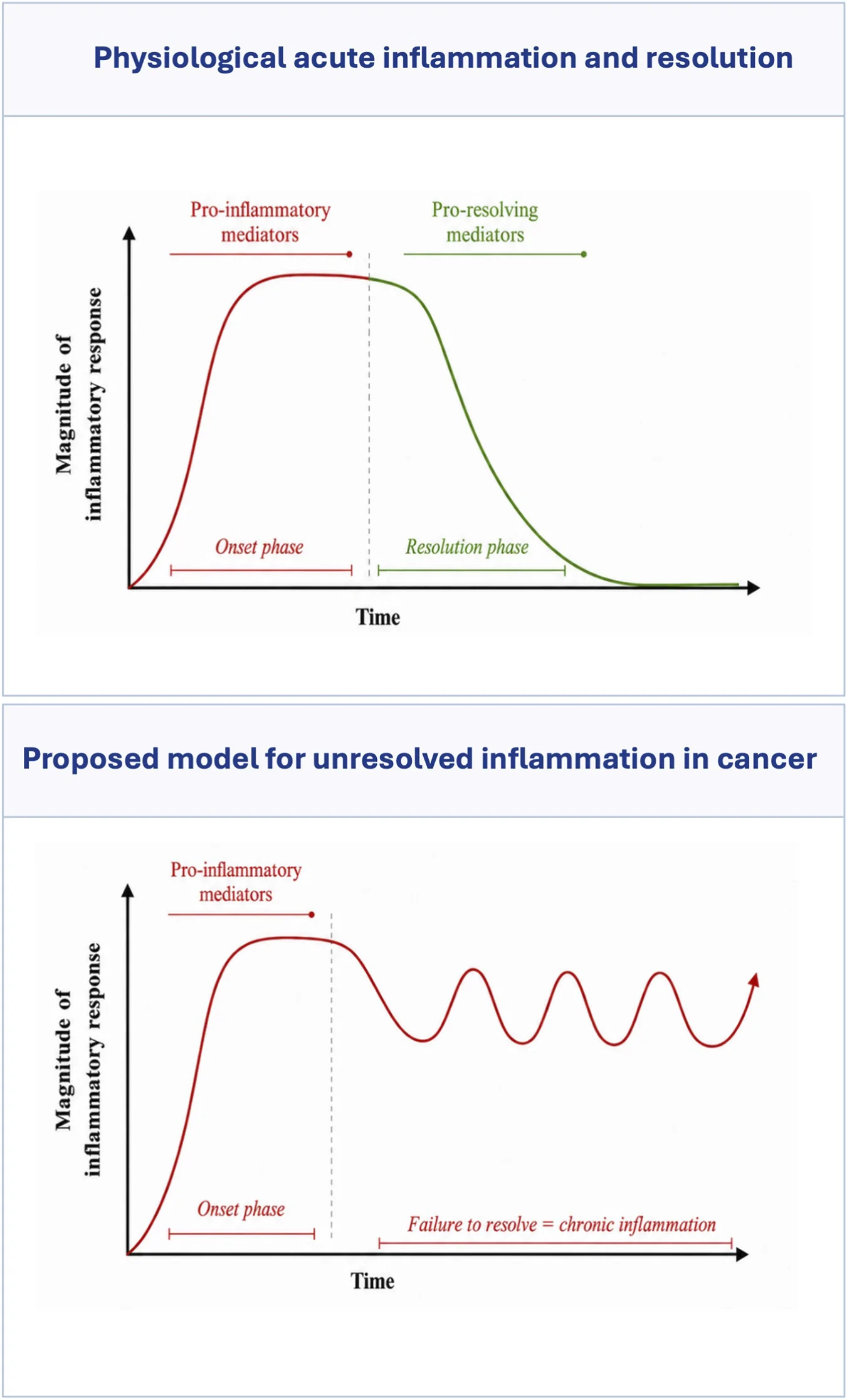
Schematic representation of the proposed model of inflammation resolution in cancer. (Upper panel) physiology: Acute tissue injury induces inflammation, followed by a class switch of lipid mediators that promotes active resolution and tissue repair. Specialized pro-resolving lipid mediators, including lipoxins, resolvins, protectins, and maresins, orchestrate this pro-resolving program. (Lower panel) cancer: In cancer, impaired resolution sustains a chronic inflammatory microenvironment, potentially contributing to tumor persistence and therapeutic resistance. Figure created with BioRender using non-generative AI assistance.

## 1 Introduction: cancer as a state of unresolved inflammation

Cancer is a major global health challenge, accounting for nearly 20 million new cases and close to 10 million deaths worldwide in 2022, and continues to represent a therapeutic challenge (Bray et al., 2024). Despite enormous progress in the knowledge about the mechanisms propelling cancer inception and progression, it appears clear that a knowledge gap still affects the way we approach such a disease. In this context, identifying biological processes that sustain tumor progression while also offering tractable therapeutic opportunities remains a priority. Some of the most therapeutically challenging features of cancer are closely linked to, and often dependent on, a chronic inflammatory milieu that actively promotes cancer cell growth and survival (Greten and Grivennikov, 2019; Hanahan and Weinberg, 2011; Mantovani et al., 2008). Several examples across different tissues and etiologies support this concept. In the gastrointestinal tract, long-standing inflammatory bowel disease increases the risk of colitis-associated colorectal cancer (Rubin et al., 2012). Chronic gastroesophageal reflux disease and Barrett’s esophagus represent a recognized inflammation-associated sequence predisposing to esophageal adenocarcinoma (Mikolašević et al., 2018; O’Sullivan et al., 2014). Another example of an established sequence from chronic inflammation to cancer is *Helicobacter* pylori-associated gastritis (Hatakeyama, 2019), and primary sclerosing cholangitis promotes cholangiocarcinoma through a dysplasia–carcinoma sequence (Taghavi et al., 2018). Additionally, chronic pancreatitis increases the risk of pancreatic ductal adenocarcinoma (Gandhi et al., 2022), and obesity-associated low-grade inflammation increases cancer risk through adipose tissue dysfunction, immune cell infiltration, and systemic pro-inflammatory signaling (Deng et al., 2016). Altogether, this supports the broader concept that inflammation drives cancer initiation, growth, progression, and metastasis (Greten and Grivennikov, 2019; Hanahan and Weinberg, 2011; Mantovani et al., 2008).

In pathophysiological conditions, inflammation is a vital adaptive response to a wide range of insults, driven by the early rise in pro-inflammatory mediators (PIMs). It is a tightly regulated phenomenon, since uncontrolled inflammation can alter tissue structure and function. Appropriate termination of inflammation is actively resolved through coordinated programs (Chiang and Serhan, 2020). A key component of this control module is the biosynthesis of specialized pro-resolving mediators (SPMs), including lipoxins, resolvins, protectins, and maresins, which arise from polyunsaturated fatty acid pathways that are shared in part with those for classical pro-inflammatory mediators, such as prostaglandins and leukotrienes (Serhan, 2014; Wang and Dubois, 2010). Persistent immune cell recruitment, stromal remodeling, and continuous production of inflammatory mediators sustain conditions reminiscent of unresolved tissue injury (Mantovani et al., 2008; Dvorak, 2015), supporting a view of tumors as “wounds that do not heal” (Dvorak, 2015). In this context, cancer-associated inflammation may follow, thus raising the question of whether and to what extent SPM-related pathways are engaged in tumors. While the cancer-promoting functions of PIMs, such as prostaglandins (PGs) and leukotrienes (LTs), are well understood (see below), therapeutic efforts to suppress these PIM pathways have yielded heterogeneous and limited results. This review examines the possible relationship between lipid mediator networks involved in the initiation and resolution of inflammation and cancer progression, with a focus on the putative role for defective programs of inflammation resolution. We summarize current knowledge of the mechanistic roles of SPMs in the tumor microenvironment and discuss emerging opportunities for restoring pro-resolving signaling (Lavy et al., 2021; Raeisi et al., 2026).

### 1.1 Novelty and scope of this review

Several excellent reviews have previously addressed the resolution of inflammation in general terms (Chiang and Serhan, 2020; Serhan, 2014; Li et al., 2026) or the pro-tumorigenic role of individual pro-inflammatory eicosanoids such as PGE2 (Wang and Dubois, 2010; Finetti et al., 2020). What has been missing is a synthesis that (i) explicitly organizes the cancer literature by the strength of the underlying evidence (direct tumor data versus evidence extrapolated from non-cancer inflammation models versus purely conceptual extension), (ii) integrates the very recent tumor-specific multi-omic and lipidomic datasets that directly measure SPM/PIM balance in human cancer tissue, and (iii) translates this synthesis into a concrete, biomarker-anchored pharmacological roadmap for oncology. The present review does not attempt to re-cover the general biochemistry of resolution, which has been comprehensively reviewed elsewhere (Chiang and Serhan, 2020; Serhan, 2014; Li et al., 2026; Dyall et al., 2022); rather, its unique contribution is to critically integrate the emerging, cancer-specific evidence for defective lipid mediator class switching (including newly available cancer lipidomic/transcriptomic datasets in colorectal cancer and malignant pleural mesothelioma) into a single translational framework, explicitly stratified by tumor type, evidence tier, and pharmacological actionability. Because the field is biologically heterogeneous - spanning biochemical, cell-based, murine, and human observational studies - we favor a structured narrative synthesis over a systematic review, for the reasons detailed in Section 1.2.

### 1.2 Methodology: literature search strategy and evidence stratification

This is a narrative, expert-based review; it does not follow a systematic review protocol (e.g., PRISMA) and does not report a formal risk-of-bias assessment for each primary study, given the biochemical and clinical heterogeneity of the underlying evidence base. To improve transparency and reproducibility, we nonetheless applied an explicit, documented search strategy. PubMed/MEDLINE and Google Scholar were searched from database inception through April 2026 using combinations of the terms “specialized pro-resolving mediator,” “resolvin,” “protectin,” “maresin,” “lipoxin,” “lipid mediator class switching,” “resolution of inflammation,” “prostaglandin E2,” “leukotriene B4,” “efferocytosis,” “tumor-associated macrophage,” combined with “cancer,” “tumor,” “tumour,” “neoplas*,” or the name of individual tumor types (e.g., colorectal, pancreatic, lung, breast, mesothelioma, head and neck). Reference lists of retrieved reviews and primary articles were hand-searched for additional relevant citations (“snowballing”), and ClinicalTrials.gov was queried for interventional studies using the terms “specialized pro-resolving mediator,” “resolvin,” or “lipoxin.” English-language original research articles, reviews, and clinical trial registrations were considered; conference abstracts without peer-reviewed follow-up and non-English records were excluded.

Because the strength of inference differs markedly across this literature, every major claim in this review is explicitly assigned to one of three evidence tiers, indicated in the text: (i) Direct cancer evidence - data obtained directly from human tumor tissue, cancer cell lines, or *in vivo* cancer models (e.g., syngeneic or genetically engineered mouse tumors); (ii) Indirect evidence - data obtained in non-cancer models of inflammation, injury, or wound healing, or in human cancer-associated but non-tumor tissue (e.g., peritumoral or premalignant tissue), and extrapolated to the cancer setting; and (iii) Conceptual/speculative - mechanistic reasoning or hypothesis-generating association (e.g., transcriptomic correlations) that has not been causally tested in a cancer model. This tiering is applied narratively throughout the text (see, in particular, Sections 3, 4, 6, and 7) and is summarized for representative studies in Table 1. We emphasize that this framework is intended to make the evidentiary basis of each statement transparent to the reader, not to grade every one of the >100 references cited exhaustively.

**TABLE 1:** Direct cancer-specific evidence for an unresolved PIM/SPM balance: study design, evidence tier, and translational implications.

Cancer type	Model/sample	SPM/PIM axis assessed	Intervention or measurement	Direction of effect	Evidence tier	Key limitations
Colorectal cancer	Human tumor vs. normal mucosa; LC-MS/MS lipidomics + single-cell/spatial transcriptomics + TCGA-COAD (Soundararajan et al., 2025)	ALOX5-LTB4 axis vs. ALOX12/ALOX15/PTGER2-4	Direct tissue lipidomics and single-cell mapping of biosynthetic enzyme expression	Reduced pro-resolving, increased pro-inflammatory lipid mediator profile in tumor vs. normal tissue	Direct (lipidomics, single-cell); indirect for bulk TCGA correlations	Cross-sectional; causality with tumorigenesis not established
Head and neck squamous cell carcinoma	TCGA bulk transcriptomic deconvolution (Mattoscio et al., 2022)	Composite D-series resolvin pathway score (ALOX5/15, FPR2, GPR18, GPR32)	Bulk mRNA-derived composite score vs. survival and immune infiltration	Higher score associated with improved survival and CD8^+^ T-cell infiltration	Indirect/inferential (transcriptomic, no direct lipid quantification)	No direct SPM measurement; associative, not causal
Malignant pleural mesothelioma	Human pleural exudates (n = 19) + scRNA-seq + TCGA-MESO; targeted LC-MS/MS of 56 oxylipins (Gatti et al., 2026)	Resolvins, protectins, maresins vs. prostaglandins; 5-gene CPS (CYP3A4, ALOX15, EPHX1, GSTM4, GPX4)	Direct lipid mediator quantification pre/post chemotherapy; LASSO-derived prognostic gene signature	Chemotherapy reduced SPMs and increased prostaglandins; low CPS/low SPM-precursor levels associated with shorter overall survival	Direct (lipidomics, prognostic modeling)	Small cohort (n = 19); observational; no causal/mechanistic validation
Non-small cell lung cancer (KRAS G12D model)	Murine model + human tumor tissue	AT-RvD1/ALX-FPR2 vs. SAA/ALOX5-driven TAN accumulation	AT-RvD1 administration; correlation of SAA/ALOX5 ratio with TAN infiltration in human tissue	AT-RvD1 reduced TAN accumulation and tumor growth; elevated SAA/ALOX5 ratio associated with pro-tumor neutrophil signature in patients	Direct (murine intervention); indirect/associative for the human correlation	Causal link in humans not established; single tumor model
Colitis-associated colorectal cancer	Murine model (aspirin intervention) (De Matteis et al., 2022)	AT-lipoxin A4, AT-resolvin D1/Alx-Fpr2	Aspirin administration; AT-SPM biosynthesis inhibition; Alx/Fpr2 knockout	Aspirin increased AT-SPMs and reprogrammed macrophage/T-cell responses; effect abrogated by AT-SPM inhibition or Alx/Fpr2 knockout	Direct (murine, mechanistic/causal)	Murine model; human translational data limited to biomarker studies

## 2 Main pathways sustaining PIM and SPM biosynthesis in pathophysiological conditions

The acute inflammatory response is dominated at its onset by the production of pro-inflammatory cytokines, chemokines, and lipid mediators, including prostaglandins (PGs), thromboxanes (TXs), and leukotrienes (LTs) (Dyall et al., 2022; Park, 2026). Following inflammatory stimulation, phospholipase A2 (PLA2) releases polyunsaturated fatty acids (PUFAs), including arachidonic acid (AA), eicosapentaenoic acid (EPA), and docosahexaenoic acid (DHA), from membrane phospholipids, making them available for downstream metabolism. These PUFAs serve as substrates for the biosynthesis of a broad spectrum of bioactive lipid mediators, whose structures and functions depend on the enzymatic pathways involved (Figure 1; Table 2). AA represents the principal precursor of classical pro-inflammatory eicosanoids and is metabolized through three major enzymatic pathways: the cyclooxygenase (COX) pathway, generating prostaglandins, thromboxanes, and prostacyclin; the lipoxygenase (LOX) pathway, particularly 5-LOX, producing leukotrienes and hydroxyeicosatetraenoic acids (HETEs); and the cytochrome P450 (CYP450) pathway, generating epoxyeicosatrienoic acids (EETs) and additional HETE metabolites (Dyall et al., 2022; Park, 2026). EPA can likewise serve as a substrate for the generation of structurally related lipid mediators, including 3-series prostaglandins and thromboxanes as well as 5-series leukotrienes through COX- and LOX-dependent pathways. Although these EPA-derived mediators generally exhibit lower inflammatory potency than their AA-derived counterparts, they help shape the inflammatory environment. They can also serve as intermediates in the generation of SPMs (Figure 1; Table 2) (Dyall et al., 2022; Park, 2026).

**FIGURE 1:**
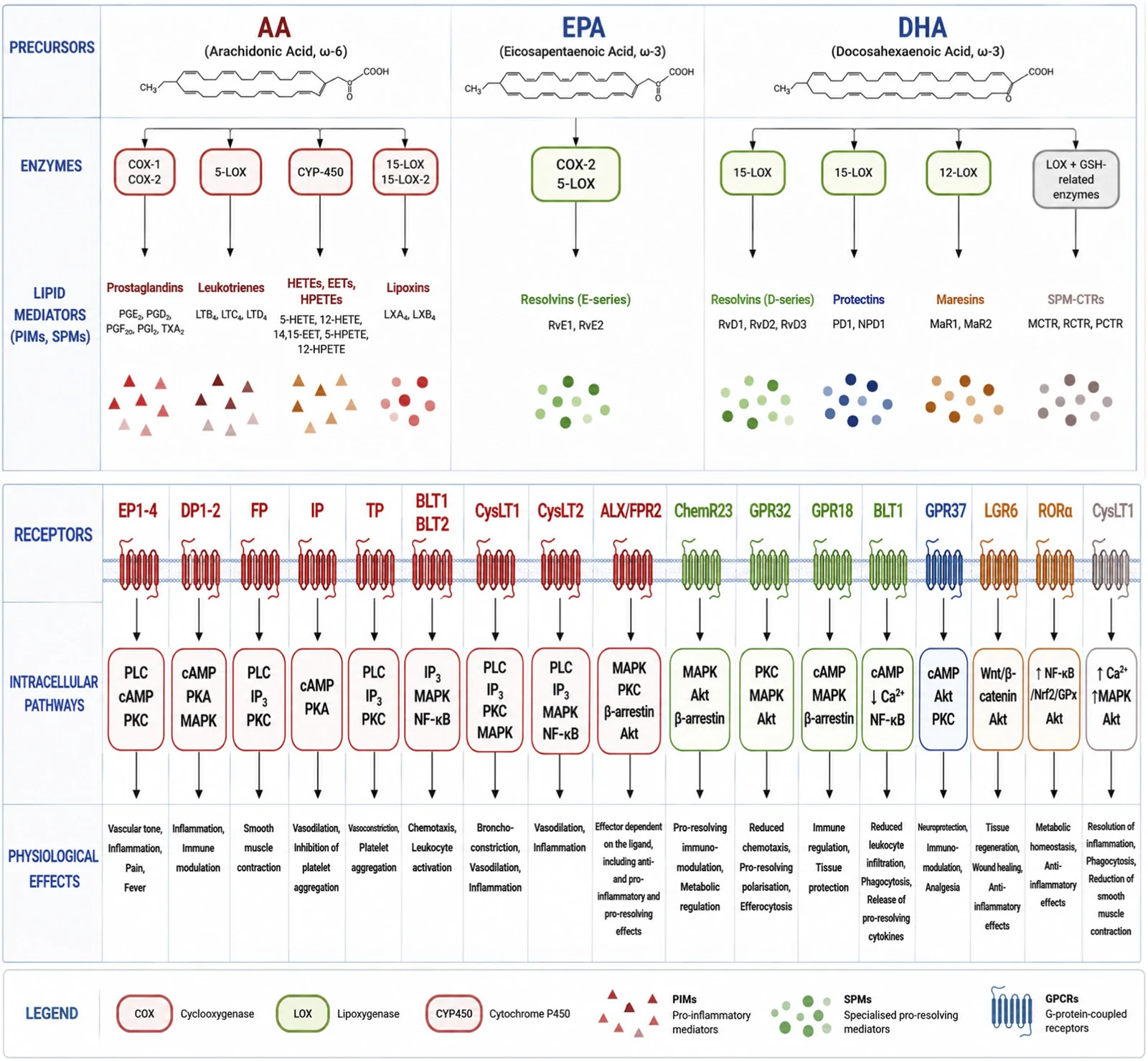
Biosynthesis and signaling of pro-inflammatory and specialized pro-resolving lipid mediators. Upper panel. Arachidonic acid (AA), eicosapentaenoic acid (EPA), and docosahexaenoic acid (DHA) are metabolized by cyclooxygenases (COX), lipoxygenases (LOX), cytochrome P450 (CYP450), and related enzymes to generate pro-inflammatory mediators (PIMs), including prostaglandins, leukotrienes, and HETEs, as well as specialized pro-resolving mediators (SPMs), including lipoxins, resolvins, protectins, maresins, and SPM-conjugates in tissue regeneration (SPM-CTRs). Lower panel. Schematic representation of the main receptors activated by these mediators, their representative intracellular signaling pathways, and their biological functions. Figure created with BioRender using non-generative AI assistance.

**TABLE 2:** Major lipid mediator families involved in inflammatory and pro-resolving signaling. The table summarizes representative mediators, biosynthetic precursors, main enzymatic pathways, biological actions, and associated receptors. SPM-CTR indicates cysteinyl-specialized pro-resolving mediators derived from the maresin pathway.

Mediator family	Representative mediators	Main precursors	Key biosynthetic enzymes	Main biological actions	Receptors
Prostaglandins	PGE_2_, PGD_2_, PGF2α, PGI_2_, TXA2	AA	COX	Vasodilation, pain, and amplification of the inflammatory response	EP1-4, DP1-2, FP, IP, TP
Leukotrienes	LTB_4_, LTC_4_, LTD_4_	AA	5-LOX	Neutrophil recruitment, vascular permeability	BLT1, BLT2, CysLT1, CysLT2
Lipoxins	LXA_4_, LXB_4_	AA	15-LOX	Resolution of inflammation, inhibition of neutrophil recruitment	ALX/FPR2, GPR32, CB1
			5-LOX		
Resolvins (E-series)	RvE1, RvE2	EPA	COX-2 LOX	Anti-inflammatory signaling, reduced leukocyte trafficking	ChemR23, BLT1
Resolvins (D-series)	RvD1, RvD2, RvD3	DHA	15-LOX	Efferocytosis, macrophage reprogramming	ALX/FPR2, GPR32, GPR18
			5-LOX		
Protectins	PD1/NPD1	DHA	15-LOX	Tissue protection, anti-inflammatory effects	GPR37, BLT1
Maresins	MaR1, MaR2	DHA	12-LOX	Macrophage-mediated tissue repair	LGR6,RORα
SPM-CTR (cysteinyl-SPM)	MCTR1, MCTR2, MCTR3	DHA	(maresin pathway) LOX + glutathione-related enzymes	Tissue regeneration, resolution of inflammation	CysLT1 (proposed)

Abbreviations: LOX, lipoxygenase; COX, cyclooxygenase; GPCR, G protein-coupled receptor; AA, arachidonic acid; EPA, eicosapentaenoic acid; DHA, docosahexaenoic acid.

Importantly, the relative contributions of these biosynthetic pathways change dynamically throughout the course of inflammation. During the transition toward resolution, a temporal shift in lipid mediator production, known as lipid mediator class switching (see the following sections), redirects PUFA metabolism from the generation of predominantly pro-inflammatory eicosanoids toward SPMs (Levy et al., 2001). Coordinated changes in the expression and activity of COX, LOX, and CYP enzymes orchestrate this process. This represents a key biochemical mechanism underlying the transition from inflammation to resolution (Levy et al., 2001). For instance, AA can also give rise to SPMs, particularly lipoxins, through sequential lipoxygenase-dependent reactions involving mainly 15-LOX and 5-LOX (Lavy et al., 2021; Li et al., 2026; Dyall et al., 2022). Concurrently, ω-3 PUFAs, including EPA and DHA, serve as substrates for the biosynthesis of additional SPM families through partially overlapping enzymatic pathways. EPA is first converted into 18-hydroxyeicosapentaenoic acid (18-HEPE) by acetylated COX-2 or CYP450 enzymes. Subsequent 5-LOX-dependent transformations generate E-series resolvins, including RvE1, RvE2, RvE3, and RvE4 (Lavy et al., 2021; Li et al., 2026; Dyall et al., 2022). In contrast, DHA undergoes initial oxygenation by 15-LOX to form 17-hydroxydocosahexaenoic acid (17-HDHA), a central branch-point intermediate that can be further metabolized through 5-LOX-dependent pathways into D-series resolvins (RvD1–RvD6) or diverted toward the biosynthesis of protectins (PDs) and maresins (MaRs) (Lavy et al., 2021; Li et al., 2026; Dyall et al., 2022) (Figure 1; Table 2). Maresin biosynthesis is initiated predominantly by 12-LOX and proceeds *via* typical 13S,14S-epoxide intermediates generated in pro-resolving macrophages.

In addition to the canonical SPM families, a subgroup of cysteinyl-containing specialized pro-resolving mediators, collectively termed conjugates in tissue regeneration (SPM-CTRs), has recently been identified (Dalli et al., 2016; de la Rosa et al., 2018; Dalli et al., 2015). This family includes maresin conjugates in tissue regeneration (MCTRs), protectin conjugates in tissue regeneration (PCTRs), and resolvin conjugates in tissue regeneration (RCTRs), all derived from DHA-generated epoxide intermediates through glutathione-dependent enzymatic reactions Figure 1 and Table 2) (Dalli et al., 2016; de la Rosa et al., 2018; Dalli et al., 2015). These mediators contribute to tissue repair and regeneration while sharing several biosynthetic features with cysteinyl leukotrienes, highlighting an intriguing biochemical link between pro-inflammatory and pro-resolving lipid mediator networks (Levy et al., 2020; Säfholm et al., 2022).

### 2.1 Lipid mediator class switching

Specialized pro-resolving mediators do not operate independently of pro-inflammatory pathways. Rather, both systems are tightly interconnected through shared biosynthetic and regulatory networks. A central mechanism linking inflammation and resolution is lipid mediator class switching, a temporally regulated process through which the production of pro-inflammatory lipid mediators is progressively redirected toward the generation of SPMs (Chiang and Serhan, 2020; Levy et al., 2001).

During the early phases of acute inflammation, arachidonic acid metabolism is dominated by the production of prostaglandins and leukotrienes. As the inflammatory response progresses, however, prostaglandins such as PGE2 and PGD2 contribute to the induction of 15-LOX, thereby promoting a shift in lipid mediator biosynthesis toward pro-resolving mediators, including lipoxins, and key SPM precursors, such as 15-hydroxyeicosatetraenoic acid (15-HETE) from AA and 18-hydroxyeicosapentaenoic acid (18-HEPE) from EPA. These metabolic changes facilitate the subsequent generation of lipoxins, resolvins, protectins, and maresins, thereby actively promoting the resolution phase of inflammation (Chiang and Serhan, 2020; Levy et al., 2001).

Thus, inflammation and its resolution are interconnected processes coordinated through shared substrates, enzymes, and regulatory circuits. The same enzymatic networks that initiate inflammatory responses progressively drive the biosynthesis of mediators that terminate inflammation and promote tissue repair, highlighting the highly regulated and self-limiting nature of acute inflammatory responses (Chiang and Serhan, 2020; Levy et al., 2001).

## 3 PIMs as drivers of tumor progression

Pro-inflammatory lipid mediators are key regulators of innate and adaptive immunity. Prostaglandins, thromboxanes, and leukotrienes are rapidly generated following tissue insult and coordinate alterations in vascular permeability, leukocyte recruitment, cytokine production, and immune cell activation (Park, 2026). The biological effects of PIMs are mediated by distinct G protein-coupled receptors (Figure 1; Table 2), whose expression patterns and signaling bias determine context-specific responses. Relevant receptor diversity allows PIMs to regulate multiple cancer-related processes, ranging from proliferation and survival to migration and immune modulation, in a stage-specific manner. (Säfholm et al., 2022; O’Callaghan and Houston, 2015). Although many bioactive lipid mediators have been identified, only a limited subset has been extensively characterized. Among these, prostaglandin E2 (PGE2) and leukotriene B4 (LTB4) are two of the best-studied examples.

### 3.1 The PGE2 axis (direct and indirect cancer evidence)

PGE_2_ is one of the best-characterized drivers of tumor-promoting inflammation (Wang and Dubois, 2010). In many cancers, PGE_2_ accumulates downstream of cyclooxygenase activity, most prominently COX-2, and signals through E-Prostanoid (EP) receptors. Four EPs (EP1–EP4) mediate downstream signaling through non-overlapping mechanisms, including Ca^2+^ signaling, cAMP-PKA activation, and engagement of the PI3K-AKT and ERK cascades (O’Callaghan and Houston, 2015), thereby promoting proliferation and survival of cancer cells. PGE_2_ induces pro-angiogenic mediators such as vascular endothelial growth factor (VEGF) and facilitates invasion through matrix-remodeling programs, including modulation of metalloproteinases (Wang and Klein, 2007). It also attenuates recruitment and function of innate immune effectors required for productive T-cell priming (Wang and Dubois, 2010). As such, genetic ablation of COX enzymes in tumor cells or pharmacological inhibition of the COX–PGE_2_ pathway restores immune surveillance and enhances responsiveness to PD-1 blockade (Zelenay et al., 2015).

EP4 has emerged as a key immunoregulatory node, thereby placing the COX-2/PGE_2_/EP4 axis at the center of a lipid-mediated program linking chronic inflammation to tumor growth and immunosuppression (Wang and Dubois, 2010; Zelenay et al., 2015; Albu et al., 2017). In colorectal (CRC) and breast cancer (BC) models, activation of the COX-PGE2 axis was also shown to increase the percentage of ALDH-high cells, which exhibit chemo-resistant traits and contribute to tumor resistance to therapy (Bellamkonda et al., 2016; Walker et al., 2022).

### 3.2 Leukotriene-driven tumor inflammation (direct cancer evidence, murine models)

Activated neutrophils and macrophages produce LTB_4_, establishing an autocrine and paracrine circuit that promotes neutrophil chemotaxis and activation (Afonso et al., 2012; He et al., 2020). Increased LTB_4_ signaling through its receptor (Leukotriene B4 Receptor 1), BLT1, was shown to increase neutrophilic inflammation, and tumor promotion was demonstrated in Kras-driven lung cancer. In such a system, exposure to crystalline silica increased LTB_4_ production, neutrophil accumulation, and tumor burden, whereas BLT1 deficiency attenuated both inflammation and tumor progression (Satpathy et al., 2015). Activation of this axis was also shown to promote NETosis - the active release of neutrophil extracellular traps (NETs) - a mechanistically important driver of metastasis (Yang et al., 2020; Cools-Lartigue et al., 2013; Fridlender et al., 2009).

### 3.3 PIM/SPM pathways and tumor metabolic reprogramming (conceptual/speculative)

Beyond their direct signaling roles, AA-, EPA-, and DHA-derived mediators intersect with pathways governing tumor metabolic reprogramming. COX-2-derived PGE2 stimulates glycolytic flux and HIF-1α stabilization in several tumor types, linking eicosanoid signaling to the Warburg phenotype. At the same time, the enzymatic competition between COX, LOX, and CYP450 branches for shared PUFA substrates implies that metabolic stress conditions (hypoxia, nutrient restriction, chemotherapy-induced lipid release) could shift flux toward either pro-inflammatory or pro-resolving branches depending on local enzyme and cofactor availability. Consistent with this, chemotherapy-induced SASP-related release of AA, EPA, and DHA has been directly linked to altered lipid mediator profiles and to metabolic adaptation in malignant pleural mesothelioma cells (Cioce et al., 2021); see also Section 6.3 and Table 1). At present, however, direct evidence that SPM-related pathways causally influence tumor metabolic reprogramming or metabolic crosstalk within the tumor microenvironment remains limited, and this connection should be regarded as an open, hypothesis-generating question rather than an established mechanism.

## 4 Established functions of the specialized pro-resolving mediators (predominantly indirect evidence, extrapolated from non-cancer inflammation models unless otherwise stated)

### 4.1 Macrophage reprogramming and efferocytosis

Macrophages constitute the major immune infiltrate of the TME and display remarkable plasticity, exhibiting pro-inflammatory (M1-like) and pro-resolving (M2-like) phenotypes (Lavy et al., 2021; Liotti et al., 2022). SPMs reprogram macrophages toward a pro-resolving phenotype, characterized by increased phagocytic activity and reduced secretion of pro-inflammatory cytokines (Lavy et al., 2021; Liotti et al., 2022) (Figure 2, top-left panel). In detail, RvD1, RvD2, RvE2, and Maresin-1 promote efferocytosis, the process by which pro-resolving macrophages recognize and remove apoptotic polymorphonuclear leukocytes (PMNs). Maresin-1, in particular, has been shown to enhance macrophage phagocytosis and the clearance of apoptotic cells, supporting a direct role for SPMs in promoting efferocytosis and inflammation resolution (Serhan et al., 2009). Efferocytosis is a crucial mechanism for clearing cellular debris and, thus, damage-associated molecular patterns (DAMPs), which would otherwise perpetuate inflammatory signaling. This implies that a deficit of SPM may cause or perpetuate inflammation (Lavy et al., 2021; Dalli and Serhan, 2012). The relevance of these mechanisms in cancer has been demonstrated in preclinical models, where resolvins enhance macrophage-mediated clearance of therapy-generated tumor cell debris, thereby limiting debris-driven inflammation and suppressing tumor growth (Sulciner et al., 2018a). Similarly, administration of pro-resolving factors enhanced macrophage efferocytosis of apoptotic cells in tumor-bearing mice, limiting cancer progression and dissemination while promoting an IFN-γ-dependent anti-tumor T-cell response (Wetzel et al., 2021). In a prostate cancer model, RvD1 and RvD2 restrained tumor growth by modulating tumor-associated macrophage polarization and reducing expression of tumor-promoting TAM markers, including CD206, VEGFA, and EGF. Notably, their anti-tumor activity was primarily attributed to macrophage reprogramming rather than to direct effects on cancer cells (Shan et al., 2020). More recently, a dendritic cell–liposome platform delivering RvE1 in colitis-associated colorectal cancer enhanced TAM phagocytic activity while simultaneously targeting tumor-infiltrating MDSCs. This strategy reversed the immunosuppressive TME and improved the therapeutic response to immune checkpoint inhibition, further supporting the potential of SPM-driven macrophage reprogramming in cancer therapy (Yi et al., 2026). The RvE1-loaded dendritic cell–liposome approach described above also reduced tumor-infiltrating Fas + MDSCs, suggesting that SPM-based strategies may target multiple immunosuppressive myeloid populations simultaneously (Yi et al., 2026).

**FIGURE 2:**
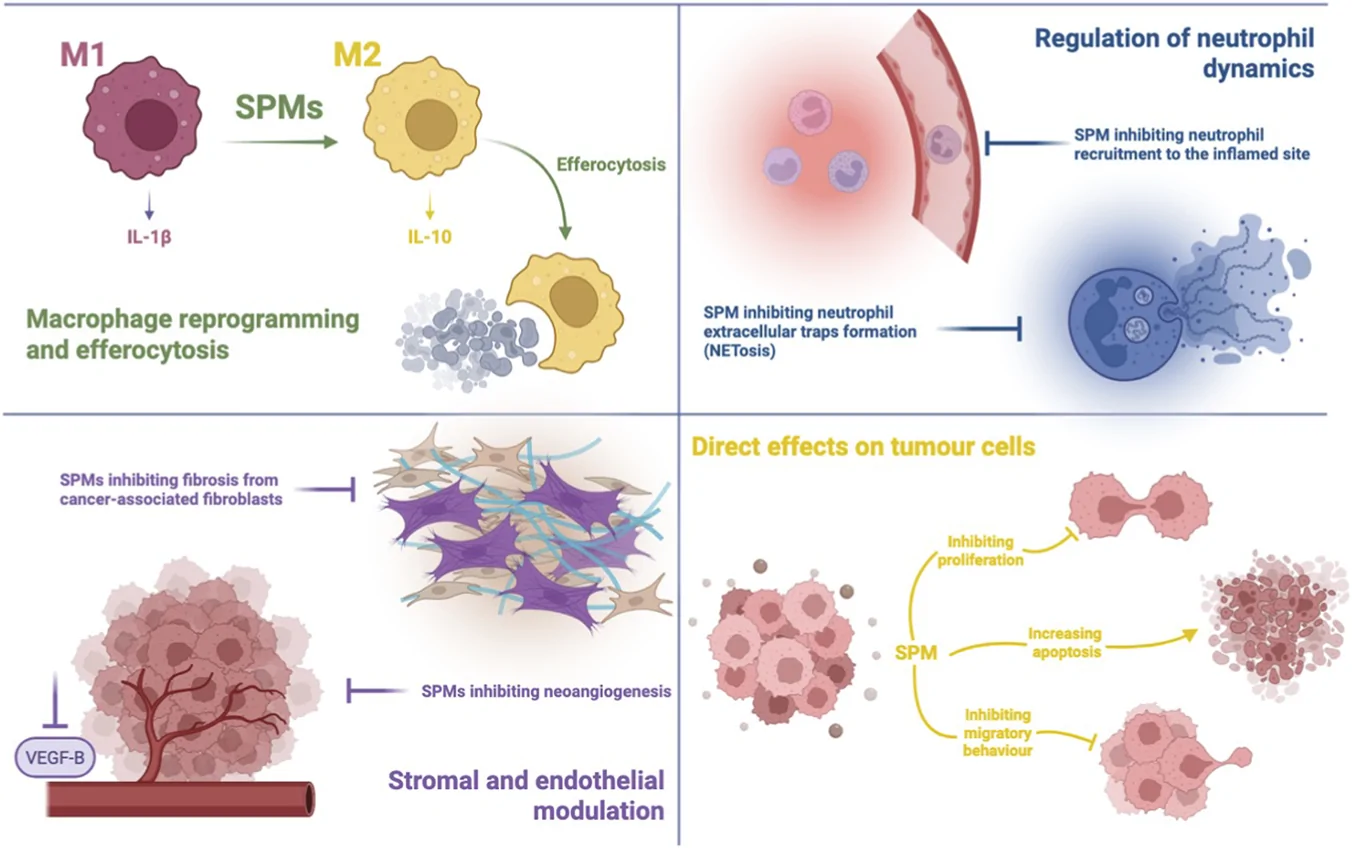
Established and emerging functions of SPMs. SPMs contribute to the resolution of inflammation by promoting macrophage reprogramming and efferocytosis, regulating neutrophil dynamics, and modulating stromal and endothelial cell activities. Furthermore, increasing evidence suggests that some SPMs may exert direct effects on tumor cells by suppressing proliferation and migratory behavior and by enhancing apoptotic pathways. Figure created with BioRender using non-generative AI assistance.

### 4.2 Regulation of immune cell dynamics

RvD1 and RvD2 have been reported to modulate tumor-associated neutrophils (TANs), thereby regulating their activation and favoring their anti-tumoral activity (Lavy et al., 2021; Li et al., 2026; Liotti et al., 2022). In HPV-positive cervical cancer models, RvD1 was shown to reprogram neutrophils and promote the recruitment of anti-tumor monocytes. Notably, neutrophil depletion impaired RvD1-mediated tumor control, providing evidence that neutrophils contribute to its anti-tumor activity (Mattoscio et al., 2021).Furthermore, RvD4 has been reported to inhibit NET formation, potentially impairing metastatic dissemination (Dyall et al., 2022; Cherp et al., 2019) (Figure 2, top right panel). SPMs can also influence the activation and differentiation of lymphocyte populations. In this context, RvD1 has been reported to increase NK-lymphocyte cytotoxicity, thereby supporting tumor immune responses (Halde et al., 2015) (Figure 2, top right panel). Lipoxin A4 (LXA4) has been reported to suppress the release of pro-inflammatory cytokines by B-lymphocytes and to attenuate the differentiation of T-lymphocytes into Tregs (Li et al., 2026; Liotti et al., 2022). In a murine colorectal cancer model, LXA4 was also shown to reshape the tumor immune infiltrate, reducing the accumulation of inflammatory neutrophils and monocytes while favoring cytotoxic lymphocytes (Liu et al., 2019). Consistent with this, in tumor-bearing mice, LXA4 reduced regulatory B-cell-mediated IL-10 production and Treg abundance, ultimately enhancing cytotoxic T-cell activity (Wang et al., 2015). Further supporting the ability of pro-resolving pathways to modulate adaptive anti-tumor immunity, De Matteis et al. (2022) showed that aspirin-induced pro-resolving signaling in colitis-associated colorectal cancer was associated with reduced PD-1 expression in CD8^+^ T cells and macrophages, thereby promoting a more active anti-tumor immune response. More recently, RvD5 has been implicated in regulating anti-tumor immunity in triple-negative breast cancer. In a 4T1 syngeneic model, activation of RvD5-associated signaling was associated with suppression of the IL-17 pathway, increased infiltration of CD4^+^ and CD8^+^ T cells, and reduced frequencies of PD-1+/LAG-3+ exhausted T cells, further linking pro-resolving signaling to the restoration of anti-tumor immune activity (Lin et al., 2026).

### 4.3 Stromal and endothelial modulation

SPMs influence additional stromal components of the TME, including fibroblasts and endothelial cells (Raeisi et al., 2026; Li et al., 2026; Liotti et al., 2022). In physiological contexts, such as wound healing, SPMs reduce fibrotic tissue deposition by fibroblasts during the proliferative phase of tissue repair. Similarly, within the cancer microenvironment, SPMs reduce the production of pro-tumorigenic mediators by cancer-associated fibroblasts (CAFs) (Liotti et al., 2022). In hepatocellular carcinoma, RvD1 was shown to disrupt CAF–cancer cell crosstalk by interfering with the paracrine activity of CAF-derived cartilage oligomeric matrix protein (COMP), thereby reducing EMT and stem-like features in cancer cells (Sun et al., 2019). Similarly, in pancreatic cancer, ALOX15-dependent production of RvD5n-3 DPA counteracted pancreatic stellate cell-induced cancer cell invasion, further supporting the ability of pro-resolving pathways to restrain tumor-promoting stromal interactions (Aguirre et al., 2023) (Figure 2, bottom left panel). SPMs also modulate the vascular compartment of the TME by disrupting endothelial function and angiogenic signaling (Lavy et al., 2021; Liotti et al., 2022; Tajbakhsh et al., 2024). Early mechanistic evidence showed that RvD1 and RvE1 exert anti-angiogenic activity, reducing inflammatory cell infiltration and VEGF expression *in vivo* (Jin et al., 2009). In cancer models, activation of inflammation-resolution pathways *via* FPR1 was shown to suppress angiogenesis and tumor growth in gastric cancer (Prevete et al., 2017). More recently, RvE1 reduced tumor vascularization in a lung cancer model, along with decreased levels of the angiogenic mediators VEGF-A, Ang-1, and Ang-2 (Kantarci et al., 2022). Further evidence of SPM-dependent regulation of tumor angiogenesis has recently emerged in gastric cancer. Activation of CB1 and CB2 receptors in gastric cancer cells promoted the production of RvD1 and LXB4, which contributed to the suppression of their angiogenic potential through SRC/MAPK-dependent signaling (Costanzo et al., 2026). At the endothelial level, the synthetic lipoxin analog ATL-1 was also shown to modulate endothelial permeability and tumor cell–endothelial interactions *via* a VEGF-dependent mechanism (Vieira et al., 2014) (Figure 2, bottom left panel). Collectively, these findings indicate that SPMs and pro-resolving pathways can remodel the tumor-supportive stroma by interfering with both CAF-derived signals and vascular programs that sustain tumor progression. The mechanisms described in this paragraph, which highlight the potential tumor-suppressive functions of SPMs in the TME, are summarized in Figure 3.

**FIGURE 3:**
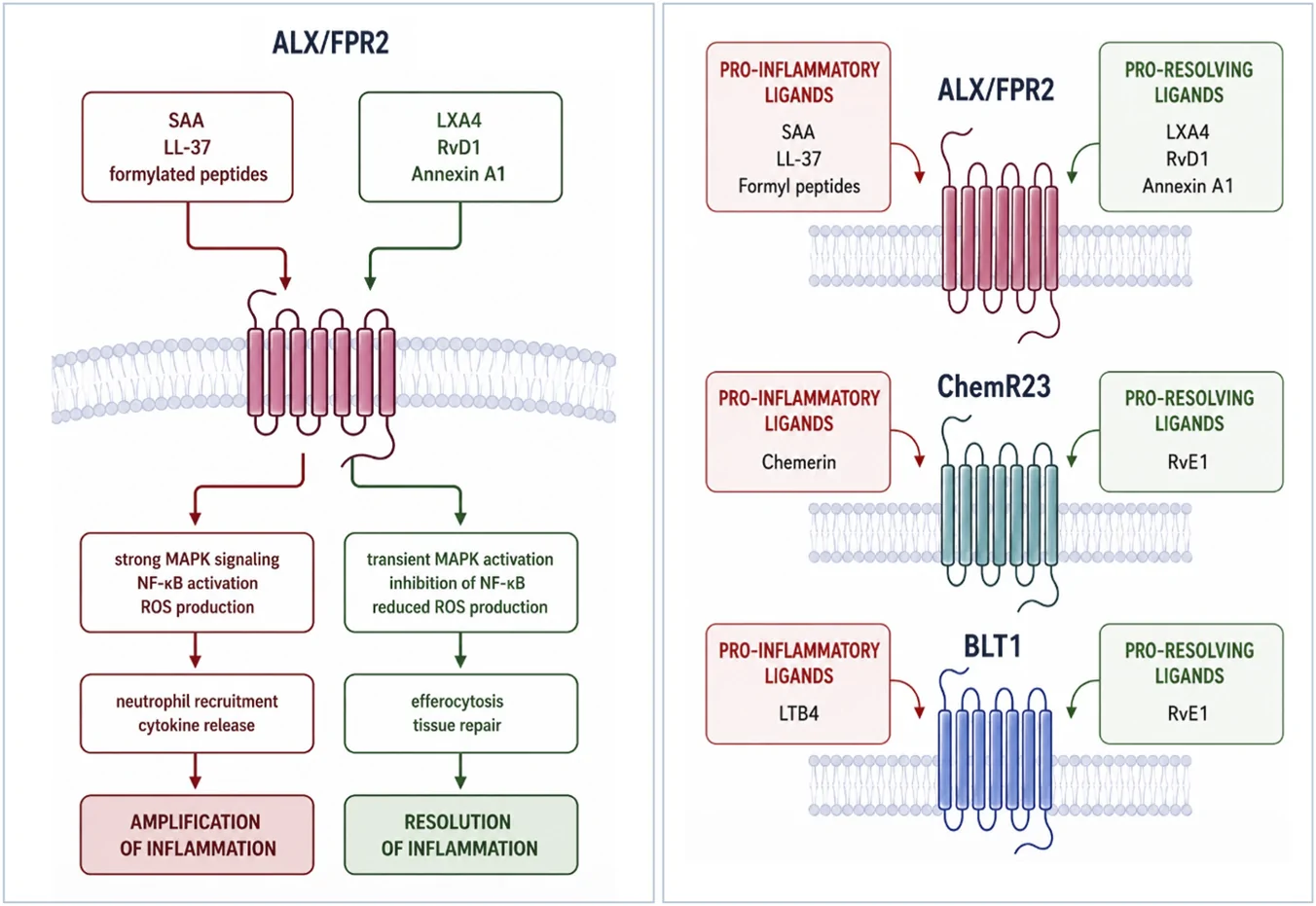
Ligand-dependent functional plasticity of ALX/FPR2, ChemR23, and BLT1. The left panel illustrates how engagement of ALX/FPR2 by pro-inflammatory or pro-resolving ligands elicits distinct signaling programs and opposite functional outcomes. The right panel summarizes representative pro-inflammatory and pro-resolving ligands acting on ALX/FPR2, ChemR23, and BLT1, highlighting the principle of biased agonism. Figure created with BioRender using non-generative AI assistance.

### 4.4 Any evidence for cell-autonomous effects of SPMs? (direct cancer cell-line evidence, *in vitro*)

Specialized pro-resolving mediators suppress cancer progression by reprogramming tumor-associated macrophages to clear cancer debris, inhibiting tumor angiogenesis, and modulating the protumorigenic functions of cancer-associated fibroblasts. These cumulative actions, which are influenced by cancer type and stage, have been observed across various tumors, including those of the oral cavity, stomach, colon, pancreas, liver, and lung, as well as melanoma, papilloma, and hematological malignancies such as leukemia (Sulciner et al., 2018b). Alongside these effects, recent experimental findings demonstrate that SPMs can directly impact cancer cells—particularly by suppressing epithelial-mesenchymal transition (EMT) and invasive behavior (Figure 2, bottom-right panel). These effects are mediated by modulation of major signaling pathways, including TGF-β/ALX/FPR2/GPR32, NF-κB/COX-2, STAT3, MAPK and NLRP3 inflammasome signaling. For example, resolvin D1 (RvD1) was shown to counteract the effects of interleukin-6 (IL-6) in the colorectal cancer cell lines SW480 and HCT15. Specifically, RvD1 diminished IL-6-induced proliferation, migration, invasion, and EMT in these cells by inhibiting IL-6/STAT3 signaling (Du et al., 2024). Beyond IL-6/STAT3 signaling, RvD1 has also been shown to directly disrupt oncogenic signaling in colon cancer cells by destabilizing aberrantly expressed c-MYC, providing further evidence of a cancer cell-autonomous action of this mediator (Zhong et al., 2018). Further, RvD1 and RvD2 inhibited TGF-β1-induced N-cadherin expression in A549, H838, H1299, and H1703 lung cancer cells at low concentrations (1–100 nM). These also suppressed the expression of zinc finger E-box binding homeobox 1 (ZEB1). As expected, resolvins attenuated the TGF-β1-induced migration and invasion in A549 cells and the morphological changes induced by TGF-β1 toward an elongated, mesenchymal-like cell shape. RvD1 is known to act *via* ALX/FPR2 and GPR32. Thus, we examined the involvement of ALX/FPR2 and GPR32 in the suppressive effects of RvD1 on TGF-β1-induced EMT of A549 cells. Gene silencing and overexpression of ALX/FPR2 and GPR32 confirmed their involvement in the effects of resolvin. blocked the action of resolvin D1 (Lee et al., 2013). The anti-EMT effect of RvD1 was also seen in nasopharyngeal carcinoma cells, where it reduced HMGB1-induced mesenchymal traits, suggesting its influence on tumor-cell plasticity is not limited to lung cancer models (Yang P. et al., 2019).

Additional research in HepG2 liver cancer cells, which express the ALX (lipoxin A4 receptor), showed that both lipoxin A4 (LXA4) and its synthetic analog BML-111 markedly suppressed hepatocyte growth factor (HGF)-induced migration and invasion. This suppression was associated with decreased expression of COX-2, MMP-2, and MMP-9, as well as inhibition of NF-κB signaling. Notably, the addition of exogenous PGE2 partially reversed these effects, highlighting the involvement of the NF-κB/COX-2 axis (Zhou et al., 2009). Comparable direct effects of LXA4 have been reported in pancreatic cancer cells, where it reduced invasive behavior by inhibiting the ROS/ERK/MMP pathway (Zong et al., 2016). A subsequent study showed that LXA4 could also reverse mesenchymal features and attenuate invasion by interfering with autocrine TGF-β1 signaling, further linking pro-resolving signaling to the control of cancer cell plasticity (Zong et al., 2017). In hepatocarcinoma cells, LXA4 and its analog were also shown to suppress EMT and migration by modulating the integrin-linked kinase axis, identifying an additional intracellular pathway through which lipoxin signaling may directly restrain malignant phenotypes (Xu et al., 2018). Another study investigated the effects of resolvin D1 on both HepG2 and PLC/PRF/5 cells. LPS treatment increased levels of inflammatory cytokines (TNF-α, IL-1β, IL-6) and promoted cell proliferation. At the same time, RvD1 treatment reduced these cytokines in a concentration-dependent manner and suppressed activation of the MAPK pathway (p-ERK, p-JNK, p-p38) (Lu et al., 2018). Direct anti-proliferative effects have also been observed in oral squamous cell carcinoma cells, with treatment with RvD1 or RvD2 reducing cancer cell proliferation *in vitro* (Ye et al., 2018). Building on these observations, Tezcan Durmus et al. recently demonstrated that RvE1 and MaR1 directly suppress NLRP3 inflammasome activation in oral squamous cell carcinoma cells. Both mediators reduced tumor cell migration and sphere formation, linking pro-resolving signaling to the regulation of inflammatory pathways that sustain aggressive cancer cell phenotypes (Durmus et al., 2026). Across most studies, SPMs exerted only modest effects on cancer cell proliferation but showed greater efficacy in modulating EMT and suppressing invasive properties (Figure 2, bottom right panel). This trend is consistent with evidence that RvD1 enhances epithelial wound healing and inhibits TGF-β-induced EMT in human alveolar epithelial cells, and reduces fibroproliferation and collagen synthesis in lung fibroblasts (Romano et al., 2019). Notably, the limited impact on proliferation contrasts with the pronounced effects of SPMs on macrophages and other TME components, suggesting context-dependent signaling mechanisms. In most experimental models, SPMs dampened responses to pro-inflammatory stimuli, such as IL-6 or TGF-β. The interplay among inflammatory mediators, PIMs, and SPMs, at the level of receptor signaling, may account for these differences and warrants further investigation. Overall, these insights highlight the therapeutic promise of SPMs in counteracting EMT-driven tumor advancement, though further research is essential to validate these effects *in vivo* and in clinical scenarios.

## 5 Receptor-level signaling and crosstalk between PIMs and SPMs (indirect evidence, non-cancer models unless otherwise specified)

### 5.1 Core GPCRs for SPM signalling

The biological activities of specialized pro-resolving mediators are primarily mediated by specific G protein-coupled receptors (GPCRs) (Figure 1; Table 2) (Serhan, 2014; Li et al., 2026). A wide variety of immune and non-immune cells, including neutrophils, monocytes, macrophages, dendritic cells, endothelial cells, epithelial cells, fibroblasts, and lymphocytes, express these receptors (Raeisi et al., 2026; Li et al., 2026; Panigrahy et al., 2021). Upon ligand binding, these receptors activate intracellular signaling cascades. Collectively, these pathways promote termination of inflammation and restoration of tissue homeostasis. Among the best-characterized SPM receptors, ALX/FPR2 plays a central role in the actions of LXA4, RvD1, RvD3, annexin A1, and other pro-resolving ligands. ALX/FPR2 is predominantly coupled to Gi proteins. This leads to inhibition of adenylate cyclase activity and modulation of downstream signaling pathways, including PI3K/Akt, ERK1/2, p38 MAPK, and NF-κB. Activation of ALX/FPR2 generally results in reduced production of pro-inflammatory cytokines, inhibition of neutrophil recruitment, and enhanced macrophage efferocytosis (Li et al., 2026; Panigrahy et al., 2021) (Figure 1; Table 2).

ChemR23 (also known as ERV1 or CMKLR1) is the principal receptor for resolvin E1 (RvE1) and is highly expressed in monocytes, macrophages, dendritic cells, endothelial cells, and natural killer cells (Ye et al., 2009; Cooray et al., 2013; Serhan et al., 2000). Like ALX/FPR2, ChemR23 primarily signals through Gi-dependent pathways. It regulates intracellular calcium mobilization, PI3K/Akt signaling, ERK1/2 activation, and suppression of NF-κB-dependent transcriptional programs (Li et al., 2026; Panigrahy et al., 2021; Laguna-Fernandez et al., 2018; Pirault and Bäck, 2018). Through these mechanisms, ChemR23 contributes to the control of leukocyte trafficking and inflammatory cytokine production (Figure 1; Table 2). BLT1 was originally identified as the high-affinity receptor for leukotriene B4 (LTB4) and can also interact with RvE1 (Li et al., 2026; Panigrahy et al., 2021). This receptor is abundantly expressed on neutrophils, monocytes, macrophages, and activated T lymphocytes. BLT1 signaling involves activation of phospholipase C (PLC), intracellular calcium flux, PI3K, MAPK, and NF-κB pathways. LTB4-induced BLT1 activation promotes chemotaxis and inflammatory amplification. In contrast, engagement of this receptor by RvE1 modulates these signaling events and helps resolve inflammation (Li et al., 2026; Panigrahy et al., 2021; Arita et al., 2007).

Additional receptors implicated in SPM signaling include GPR32 (DRV1), a receptor for RvD1, RvD3, and RvD5. GPR18 (DRV2) mediates the actions of RvD2. GPR37 has been identified as a receptor for protectin D1. LGR6 has been proposed as a receptor for MaR1 (Krishnamoorthy et al., 2010; Chi et al., 2017; Im, 2020). Several SPMs have also been reported to regulate transcriptional programs through nuclear receptors. These include retinoic acid receptor-related orphan receptor-α (RORα) (Im, 2020). The signaling mechanisms downstream of these receptors remain less extensively characterized than those of ALX/FPR2, ChemR23, and BLT1. However, the available evidence suggests convergence toward pathways that regulate leukocyte activation, cytokine production, phagocytosis, efferocytosis, and tissue repair (Figure 1; Table 2).

### 5.2 SPM–PIM cross-talk: shared receptors, distinct cellular responses

Many SPM receptors function as dynamic molecular switches that combine diverse extracellular signals and determine whether inflammatory responses are amplified or actively resolved.

The FPR2/ALX receptor FPR2 can bind both pro-inflammatory ligands, such as Serum Amyloid A (SAA), LL-37, and formylated peptides, and pro-resolving ligands, including LXA4, RvD1, and Annexin A1 (Serhan, 2014; Ye et al., 2009), thus activating different intracellular signaling pathways depending on the bound ligand, a phenomenon known as biased agonism. Pro-inflammatory ligands preferentially induce robust G protein–dependent signaling, leading to activation of MAPKs and NF-κB, and to ROS production, ultimately promoting neutrophil recruitment, cytokine release, and the amplification of inflammation. In contrast, pro-resolving ligands promote a more regulated, β-arrestin-associated signaling profile, characterized by controlled PI3K/Akt and transient ERK activation, NF-κB inhibition, and reduced ROS production, consequently supporting efferocytosis, tissue repair, and resolution of inflammation (Chiang and Serhan, 2020; Cooray et al., 2013; Krishnamoorthy et al., 2010; Chiang and Serhan, 2020; Aguirre et al., 2023; Kantarci et al., 2022).

Similar functional features have also been described for other receptors involved in the resolution of inflammation, particularly ChemR23 and BLT1. ChemR23 (also known as ERV1/CMKLR1) binds the pro-resolving mediator Resolvin E1, which promotes anti-inflammatory and pro-resolving responses through regulated PI3K/Akt and ERK signaling, as well as NF-κB inhibition. However, ChemR23 also recognizes chemerin-derived peptides, which can induce chemotactic and pro-inflammatory responses associated with stronger G protein–mediated signaling and activation of MAPKs and inflammatory pathways (Chiang and Serhan, 2020; Arita et al., 2007). Similarly, the leukotriene B4 receptor BLT1 is classically associated with pro-inflammatory signaling mediated by LTB4, leading to neutrophil recruitment, calcium mobilization, MAPK activation, and amplification of inflammation. Interestingly, the pro-resolving mediator RvE1 can also interact with BLT1, acting as a functional antagonist or partial agonist to dampen inflammatory signaling and promote resolution (Arita et al., 2007). (Figure 3, right panel). Taken together, this evidence indicates that receptors involved in inflammation are not simply “on/off” inflammatory switches but rather dynamic signaling platforms that integrate distinct extracellular cues and generate ligand-specific intracellular responses through biased agonism and signaling crosstalk.

## 6 SPM biosynthesis as a dynamic and context-dependent process

### 6.1 Local SPM production: the tumor microenvironment (mixed direct/indirect evidence)

During the resolution phase of inflammation, lipid mediator production is tightly coordinated in both space and time and involves contributions from multiple cell populations, rather than being generated exclusively by a single cell type. SPMs arise from active interactions among leukocytes, endothelial cells, epithelial cells, stromal cells, and tissue-resident cells. As a result, the local balance between PIMs and SPMs is determined not only by the biosynthetic capacity of individual cells but also by the organization of the tissue microenvironment and the communication between neighboring cell populations (Chiang and Serhan, 2020) (Figure 4). This multicellular coordination is exemplified by transcellular biosynthesis, in which lipid intermediates generated by one cell type are transferred to adjacent cells expressing complementary enzymatic machinery and further converted into bioactive mediators, hence expanding the repertoire of locally produced mediators (Serhan et al., 2000; Sala et al., 2010; Fabre et al., 2002; Serhan and Sheppard, 1990). For example, leukocyte-derived intermediates can be metabolized by platelets, endothelial cells, or tissue-resident cells to generate lipoxins and other SPMs. Early experimental studies demonstrated that interactions between neutrophils and platelets promote the transcellular conversion of leukocyte-derived leukotriene A4 (LTA4) into lipoxins through platelet 12-lipoxygenase activity, providing direct evidence for multicellular SPM biosynthesis. In this context, platelet-derived 12-LOX and endothelial 15-LOX play important roles in converting leukocyte-derived intermediates into pro-resolving mediators. Such cooperative biosynthetic pathways increase the efficiency and spatial precision of SPM production, assuring that resolution signals are generated at sites where they are most needed (Serhan and Sheppard, 1990; Papayianni et al., 1995; Edenius et al., 1991). A growing tumor is a dynamic mixture of altered cells, immune cells, and tumor-associated normal tissue cells; together, these components create an extremely complex, rapidly evolving microenvironment. Cell density, the primary site of the tumor, hypoxia gradients, elasticity, and local tissue metabolism all contribute to intratumor heterogeneity and provide the basis for relevant topographical and functional regional differences within the same tumor mass (de Visser and Joyce, 2023). Regarding hypoxia, there is a two-way relationship between hypoxia and PIM modulation: hypoxia promotes the release of PIMs (TNF-alpha and IL-1beta), which, in turn, may activate HIF-1alpha-related pathways (Malkov et al., 2021), hence amplifying the effect. This is likely to influence the distribution of PIMs, both qualitatively and quantitatively (Figure 4). On the other hand, mild physiologic hypoxia (1% O2), as encountered in the acute inflammatory response, bone marrow, and spleen, induces lipolysis-dependent biosynthesis of specialized pro-resolving mediators, including resolvins, protectins, maresins, and lipoxins (Norris et al., 2019). To further complicate the scenario, adaptive mechanisms to exogenous stressors, such as chemotherapy, may play an important role. We and others have shown that in mesothelioma and ovarian cancer tissues, chemotherapy-induced release of AA drives and activates pathways of therapy resistance, promoting the emergence of chemoresistant cells (Cioce et al., 2021; Yang J. et al., 2019) (Figure 4).

**FIGURE 4:**
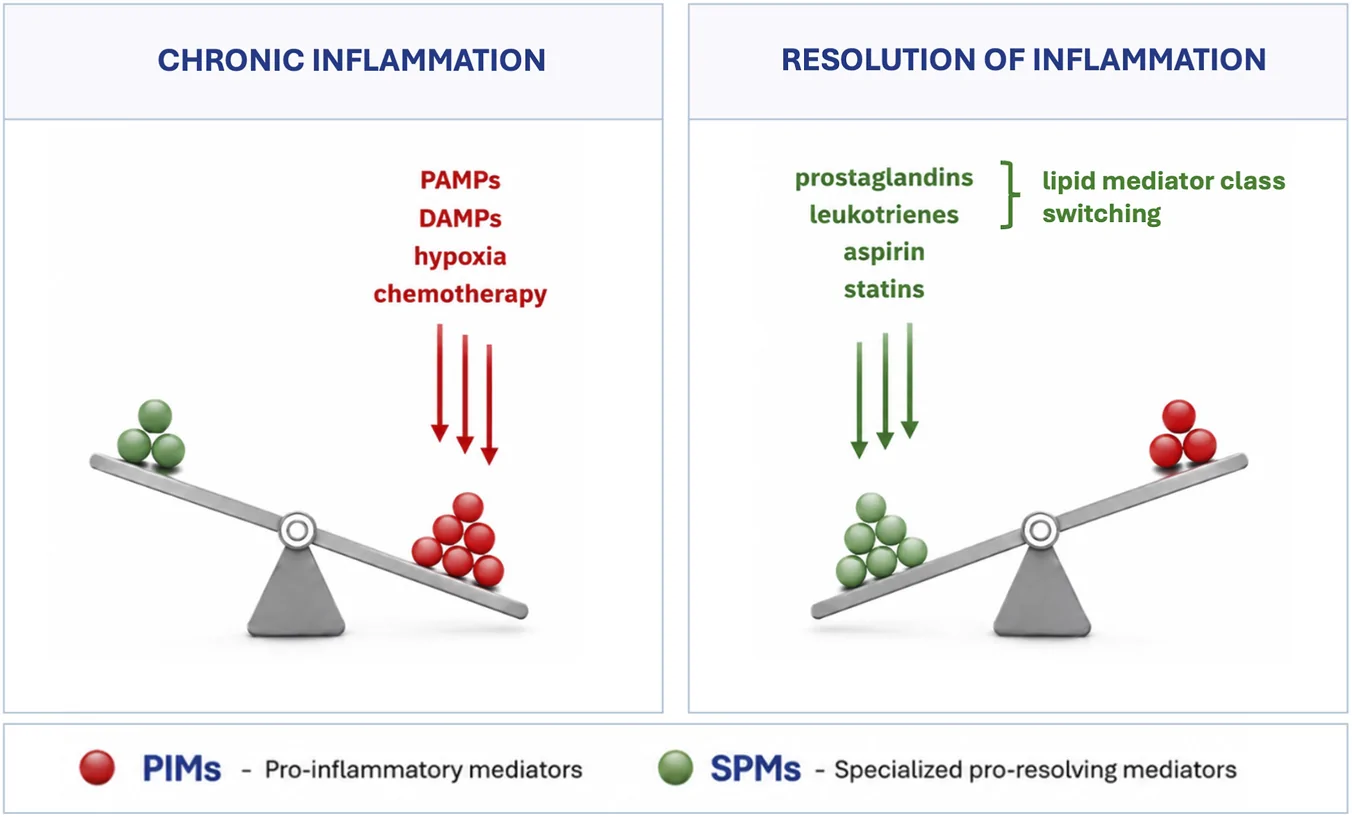
Dynamic balance between pro-inflammatory mediators and specialized pro-resolving mediators. Persistent inflammatory stimuli, including PAMPs, DAMPs, hypoxia, and chemotherapy-induced tissue damage, favor PIM predominance and chronic inflammation (left). In contrast, lipid mediator class switching and enhanced SPM biosynthesis, promoted by aspirin and statins, shift the balance toward inflammation resolution (right). Figure created with BioRender using non-generative AI assistance.

### 6.2 Local SPM production: cancer cells (direct cancer evidence)

Determinants of lipid mediator production include the expression and activity of key biosynthetic enzymes, including COXs, LOXs, and CYPs, as well as downstream enzymes involved in lipid mediator processing. Changes in the expression, localization, or activity of these enzymes may profoundly alter the repertoire of mediators generated within a tissue (Serhan, 2014; Li et al., 2026; Panigrahy et al., 2021). Expression of SPM-biosynthetic enzymes appears heterogeneous across tumor types and experimental settings (Raeisi et al., 2026; Soundararajan et al., 2025).

Tumor cells can express components of SPM biosynthetic pathways, including ALOX5, ALOX12, and ALOX15, and differential regulation of these enzymes may contribute to the defined lipid mediator profiles observed in tumors compared with healthy tissues. For example, in colitis-associated colorectal cancer models, suppression of 15-LOX-1/ALOX15 was associated with enhanced IL-6/STAT3 signaling and increased tumorigenesis, whereas restoration of 15-LOX-1 expression reduced IL-6 expression, STAT3 activation, and tumor-promoting inflammatory signaling (Mao et al., 2015).

### 6.3 Transcriptomic and lipidomic signatures of failed resolution programs in cancer (direct cancer evidence)

Beyond candidate-gene approaches, integrated multi-omic datasets now provide direct, tumor-derived evidence of impaired resolution. In colorectal cancer, a recent study combining LC–MS/MS lipidomics, single-cell and spatial transcriptomics, and TCGA-COAD analyses recorded an “unresolved” pro-inflammatory state, characterized by enrichment of the ALOX5–LTB4 axis, reduced expression of lipid class-switching genes (ALOX12, ALOX15, IL4, IL13), and decreased expression of several prostaglandin receptors (PTGER2/3/4) in tumors relative to normal mucosa (Soundararajan et al., 2025). This study directly measured lipid mediators in tumor tissue by mass spectrometry rather than inferring SPM status from mRNA alone, and further localized reduced SPM-biosynthetic capacity to malignant epithelial cells using single-cell and spatially resolved transcriptomics, thereby providing tumor cell-intrinsic, direct evidence for defective pro-resolving competence, in addition to the inferential transcriptomic layer. On this basis, the authors proposed that defective prostaglandin-dependent lipid class switching may limit the generation of endogenous pro-resolving mediators and contribute to persistent tumor-associated inflammation (Soundararajan et al., 2025).

In parallel, TCGA-based analyses in head and neck squamous cell carcinoma identified associations between a higher composite D-series resolvin pathway score, integrating biosynthetic enzymes (ALOX5/ALOX15), receptors (FPR2, GPR18, GPR32), and degradative enzymes, and improved clinical outcome, as well as enhanced CD8^+^ T-cell-related anti-tumor immunity (Mattoscio et al., 2022). Because this analysis relies on bulk transcriptomic deconvolution rather than direct lipid mediator quantification, it remains, by our evidence-tiering framework (Section 1.2), an indirect/inferential association rather than direct proof of altered SPM biosynthesis. However, it is directionally consistent with the colorectal cancer lipidomic data above.

### 6.4 Direct lipidomic evidence and a prognostic SPM-based signature in malignant pleural mesothelioma (direct cancer evidence)

The most direct evidence to date linking impaired resolution to clinical outcome comes from a targeted metabololipidomic study of pleural exudates from patients with malignant pleural mesothelioma (MPM), a paradigmatic inflammation-driven, chemoresistant cancer (Gatti et al., 2026). Using LC-MS/MS, this study directly quantified 56 oxylipins - 22 pro-inflammatory mediators and 34 SPMs derived from AA, EPA, and DHA - in the pleural fluid of chemotherapy-naïve and chemotherapy-treated patients. Chemotherapy exposure was associated with a net increase in prostaglandins (PGE2, PGD2, PGF2α) and a reduction in several SPMs, including resolvin D1, resolvin E1, maresin 2, and the protectins PDX and AT-PD1, indicating that cytotoxic treatment itself can suppress pro-resolving lipid mediator output in human tumor exudates (Gatti et al., 2026). Exploiting available single-cell RNA-sequencing data of matched tumor tissue further showed that mesothelioma cells express lower levels of several SPM-biosynthetic enzymes than adjacent diploid mesothelial cells, suggesting an intrinsic, transformation-associated defect in SPM biosynthetic capacity that is exacerbated, rather than caused, by chemotherapy (Gatti et al., 2026).

Critically for translational purposes, this study derived a five-gene composite prognostic score (CPS; CYP3A4, ALOX15, EPHX1, GSTM4, GPX4) from lipid-metabolism/oxidative-stress genes in the TCGA mesothelioma dataset, using LASSO regression with bootstrap resampling. Patients with a low CPS (indicating reduced SPM-pathway gene expression) had a significantly higher risk of death (adjusted hazard ratio 0.61 for high versus low CPS, favoring high CPS; time-dependent AUC 0.66–0.78 at 12–36 months) and showed enrichment of Wnt signaling and a higher proportion of M1 macrophages, independent of treatment status, histological subtype, and pathological stage (Gatti et al., 2026). Furthermore, four SPM precursors (15-HEPE, 18-HEPE, 20-HDOHE, 7-HDOHE) together with RvE2 and protectin DX correlated significantly with overall survival across the same patient cohort (Gatti et al., 2026). Together, these data provide direct, patient-derived lipidomic and prognostic evidence - rather than purely conceptual extrapolation - that reduced SPM output characterizes more aggressive, chemoresistant MPM, and they nominate a concrete, testable panel of candidate biomarkers (tissue/plasma SPM precursor levels, RvE2, protectin DX, and the CYP3A4/ALOX15/EPHX1/GSTM4/GPX4 gene signature) for future patient stratification (see also Section 7.5; Table 1). As acknowledged by the authors, this study remains observational, is based on a limited patient cohort (n = 19 for lipidomics), and does not establish causality between defective resolution and chemoresistance; mechanistic validation is required (Gatti et al., 2026).

Although the colorectal and mesothelioma datasets differ in tumor biology and in the depth of direct lipid quantification, they converge on a common pattern - reduced SPM/SPM-pathway output associated with a more inflamed, less favorable tumor state - across two histologically and etiologically distinct cancers, which strengthens the generalizability of the “failed resolution” concept while still falling short of establishing a unifying causal mechanism.

No validated SPM-directed cancer therapy currently exists in clinical practice; the table above summarizes mechanistic and prognostic evidence supporting the biological rationale for such strategies, not evidence of clinical efficacy.

## 7 Preclinical evidence for antitumor activity of SPM-based strategies

### 7.1 A tale of two faces: statins and aspirin, new, “old” inspiring models? (mixed direct/indirect evidence, predominantly murine)

Statins and aspirin converge on a distinct pro-resolving axis of inflammation by increasing SPM levels (Spite and Serhan, 2010), and evidence indicates that this mechanism can limit pathological inflammation and may underlie their anticancer properties. Statins, widely used HMG-CoA inhibitors, have been shown to raise the pro-resolving lipid lipoxin A4 (Campos-Estrada et al., 2015; Birnbaum et al., 2006) (Figure 4). Molecular docking studies have shown the affinity of statins for 15-LoX and CoX-2. This is biologically relevant. For example, Simvastatin-induced LXA4 was shown to engage the FPR2 receptor and attenuate downstream p38 MAPK signaling, promoting neutrophil apoptosis and reducing neutrophil brain infiltration, thereby attenuating tissue injury in models of intracerebral hemorrhage (Zhang et al., 2022). Long-term low-dose aspirin may reduce the risk of developing lung adenocarcinoma (Rothwell et al., 2011; Yu et al., 2022; Gebauer et al., 2021), although the mechanistic basis of this association is poorly understood. Aspirin’s anti-inflammatory actions are elicited by blocking COX-2 synthesis of prostaglandins, which, as mentioned above, are implicated in angiogenesis, tumor metastasis, and suppression of antitumor immunity (Cha and DuBois, 2007). The latter has long been known as the main mechanism of aspirin’s anticancer action. However, the fact that newer classes of COX-2 inhibitors, such as those that failed to provide any survival advantage in NSCLC and other cancer settings, evokes an intriguing explanation (Edelman et al., 2017). Aspirin (acetylsalicylic acid) is unique among common non-steroidal anti-inflammatory drugs (NSAIDs) in that it is the only one that covalently modifies and inhibits COX-2 by acetylating its active site. At the same time, other, more recent NSAIDs (such as ibuprofen and naproxen) bind to COX-2; aspirin specifically transfers its acetyl group irreversibly to the hydroxyl side chain of Ser516 within the COX-2 active site. This specific acetylation of COX-2 alters the enzyme’s function, enabling it to produce Aspirin-triggered SPMs (Figure 4).

In a KRAS G12D lung adenocarcinoma mouse model, aspirin-triggered resolvins such as AT-RvD1, which act through ALX/FPR2 to suppress neutrophil trafficking instigated by ALX/FPR2 ligands such as serum amyloid A, lower the neutrophil-to-lymphocyte ratio and enhance macrophage-mediated clearance of tumor debris, with attendant reductions in tumor growth in preclinical lung adenocarcinoma models. TANs can support tumor growth by suppressing cytotoxic lymphocytes. AT-RvD1 is an eicosanoid that can antagonize neutrophils. In similar work, reduced ALOX5 expression in human lung adenocarcinomas and a strong correlation between an elevated SAA/ALOX5 ratio and TAN accumulation were reported, linking impaired endogenous SPM biosynthesis to a pro-tumor neutrophil signature. In addition, AT-RvD1 prevented TGF-β1-induced EMT in lung cancer cells (A549 cells) (Liu et al., 2016) and was more recently shown to attenuate the cigarette smoke extract-induced EMT in bronchial epithelial cells (BEAS-2B) (de Oliveira et al., 2024).

In line with this, administration of DHA in the presence of ASA-acetylated COX-2 increased antiangiogenic effects by modulating endothelial cell (EC) proliferation, motility, and capillary-like tube formation, and this correlated with EC biosynthesis of pro-resolving lipid mediators (Vara-Messler et al., 2025).

Finally, in a murine model of inflammation-associated colorectal cancer, aspirin increased colonic concentrations of AT-lipoxin A4 and AT-resolvin D1, downregulated PD-1 expression on macrophages and CD8^+^ T cells, and reduced tumor burden; these protective, immune-reprogramming effects were abrogated by pharmacological inhibition of AT-SPM biosynthesis or by genetic knockout of the AT-SPM receptor Alx/Fpr2, and were substantially recapitulated by direct administration of AT-SPMs, providing direct, causal (rather than merely correlative) evidence that AT-SPM production mediates part of aspirin’s chemopreventive activity in this model (De Matteis et al., 2022). AT-SPMs may represent the first practical examples of therapeutically raising endogenous SPMs to treat cancer. This resolution-based strategy terminates harmful inflammation while preserving or even enhancing anti-tumor immunity. Together, these data support a unifying concept that pharmacologic elevation of SPMs can actively terminate inflammation while preserving or even enhancing key anti-cancer immune functions, in contrast to broad anti-inflammatory strategies that blunt pro-inflammatory cascades and risk impairing cytotoxic immune responses.

### 7.2 Potential antitumor activity of SPM-based strategies (preclinical and early clinical/observational evidence)

Although the biological rationale for specialized SPM-based strategies in oncology is compelling, their clinical translation remains in its early stages. At present, the translational funnel remains narrow, driven predominantly by mechanistic preclinical evidence rather than by efficacy-oriented clinical development. As mentioned above, Aspirin-triggered lipoxin A4 analogs have been reported to shift macrophage polarization toward a less tumor-supportive phenotype, thereby contributing to tumor control. Likewise, resolvin D1 and D2 have been shown to suppress tumor growth and inflammatory signaling through immunomodulatory mechanisms (Shan et al., 2020; Simões et al., 2017). In prostate cancer, this activity has been traced specifically to tumor-associated macrophage (TAM) reprogramming: in Pten-null mice, a genetic model of prostate cancer, a diet enriched in DHA slowed disease progression relative to an ω-6-enriched diet, and RvD1/RvD2 reduced the expression of TAM polarization markers (CD206, VEGFA, EGF) shared by pro-inflammatory M1-like and IL-4-driven M2a-like TAM subsets, suggesting that resolvins act on prostate tumors predominantly by reprogramming the macrophage compartment rather than through a direct, tumor cell-autonomous mechanism (Shan et al., 2020). Lipoxin A4 biomimetics, such as NAP1051, have demonstrated antitumor activity in colorectal cancer models, associated with reduced neutrophil infiltration, diminished accumulation of myeloid-derived suppressor cells, and decreased NETosis. (Dong et al., 2021). Additional pro-resolving dietary formulations, such as SuperMApo, further support the concept that pharmacologic engagement of endogenous resolution circuits may enhance antitumor immunity and restrain cancer progression (Wetzel et al., 2021). Notably, in this context, the ChemR23 axis: Lavy et al. (2021) showed that agonistic anti-ChemR23 engagement reprograms M-CSF-derived and TAM-like macrophages toward a less inflammatory and less immunosuppressive phenotype, while reducing metastatic dissemination and prolonging survival in a resected triple-negative breast cancer model. Data from human cancer settings have recorded altered circulating lipoxin and resolvin profiles in patients with pancreatic cancer (Blogowski et al., 2021). The clinical trial landscape is pretty limited: An interesting study is the NCT06887673, which is currently focusing on supplementing cancer patients (Colorectal Cancer, Sarcoma, Brain Tumors, Endometrial Cancer, Ovarian Cancer) with a leukotriene inhibitor, together with almonds/almond oil and SPM-containing dietary supplements, in addition to the standard of care. Endpoints of the study will encompass lipid mediator concentrations, tumor-associated macrophage reprogramming, and immune cell function, rather than conventional oncological efficacy endpoints such as objective response, progression-free survival, or overall survival (University of South Florida, 2026).

### 7.3 Combination strategies (preclinical evidence)

At present, a translational rationale for SPM-based therapeutics in oncology could be in combinatorial settings. Preclinical studies showed that resolvins suppressed debris-stimulated tumor progression and enhanced the activity of standard anticancer agents, with additive or synergistic effects reported when combined with gemcitabine, cisplatin, cetuximab, and erlotinib (Sulciner et al., 2018a). A specific example of such synergy comes from lung cancer, where resolvin E1 combined with cisplatin enhanced tumor growth inhibition compared with either agent alone, an effect linked to reduced expression of pro-inflammatory and pro-angiogenic factors (COX-2, NF-κB, VEGF-A, angiopoietin-1/2) and to reduced tumor-associated macrophage and neutrophil infiltration in a KRAS-driven lung adenocarcinoma model, together with a lowered neutrophil-to-lymphocyte ratio (Kantarci et al., 2022). Potential synergy with targeted therapies may also extend beyond tumor-cell intrinsic signaling to include stromal and macrophage reprogramming. In human papillomavirus (HPV)-positive cervical cancer, RvD1 reduced tumor cell proliferation both *in vitro* and in mouse and human models of HPV-positive disease; pharmacological depletion of neutrophils *in vivo* demonstrated that this effect depended on a neutrophil-driven recruitment of anti-tumor monocytes rather than on a direct action on tumor cells, and mining of TCGA immune-cell infiltration data linked higher intratumoral monocyte levels to a stronger immune response and to better clinical outcomes in cervical cancer patients (Mattoscio et al., 2021). Lipoxin A4 inhibited pancreatic stellate cell differentiation into cancer-associated fibroblasts and attenuated pro-tumorigenic stromal activation in pancreatic cancer models (Schnittert et al., 2018). Likewise, lipoxin signaling has been shown to re-educate M2-like tumor-associated macrophages toward a less tumor-permissive phenotype (Simões et al., 2017). Taken together, these data suggest that SPM analogs or biomimetics may be attractive partners for targeted or microenvironment-directed regimens, especially in desmoplastic or myeloid-rich tumors; however, this positioning remains preclinical and should be interpreted with caution.

### 7.4 Stabilized SPM analogs in translational oncology (preclinical/early-phase clinical evidence, non-oncology indications)

A major obstacle to the therapeutic development of SPMs is that native mediators, although highly bioactive, are chemically labile and rapidly inactivated by enzymes *in vivo*. This liability has driven the development of stabilized analogs and biomimetic compounds designed to preserve pro-resolving receptor signaling while improving metabolic resilience and pharmaceutical tractability. In the E-series resolvin pathway, RvE1 is rapidly converted to less active metabolites, and medicinal-chemistry efforts have generated metabolically stabilized RvE1 analogs, including 19-(p-fluorophenoxy)-RvE1, that retain potent anti-inflammatory and pro-resolving actions despite reduced susceptibility to oxidative inactivation (Arita et al., 2006). Parallel work in the lipoxin field led to benzo-lipoxin A4 analogs, in which the unstable tetraene chain is replaced by a benzo-fused scaffold, thereby improving thermal and metabolic stability while preserving ALX/FPR2-linked bioactivity and pro-resolving function (Sun et al., 2009). From a translational standpoint, these compounds are more attractive than native SPMs as oncology candidates because they offer better manufacturability, formulation potential, and dosing feasibility. Importantly, the feasibility of this strategy has already been demonstrated in humans outside oncology, where a benzo-lipoxin A4 formulation (BLXA4) showed a favorable safety profile and biologic activity in a randomized Phase 1 trial, providing an important proof of concept for repurposing stabilized-resolution agonists for cancer-oriented drug development (Chiang and Serhan, 2020; Hasturk et al., 2021). Of course, translational priorities within this context may include defining dose and timing that maximize AT-SPM biosynthesis without off-target effects; developing and validating biomarkers (tissue and plasma SPM profiles, ALOX5 expression, SAA/ALOX5 ratio, NLR, ALX/FPR2 levels, EMT markers); and testing combinations with checkpoint inhibitors or other immunotherapies to assess synergy or interference. Rigorous early-phase clinical trials with mechanistic endpoints are required to determine whether AT-SPMs can move from promising preclinical agents to validated cancer therapeutics.

### 7.5 Delivery strategies and pharmacology

Recent pharmaceutical work emphasizes that native SPMs face major development barriers, including rapid metabolic degradation, low bioavailability, and physicochemical instability, all of which complicate dose selection and pharmacokinetic control (de Azevedo et al., 2026). For this reason, delivery strategies have shifted toward approaches that protect the lipid mediator and prolong local activity, particularly nanoparticle-based systems and other controlled-delivery platforms. Proof of principle for this concept has already been demonstrated with pro-resolving nanomedicines containing lipoxin biomimetics, which have improved tissue repair and regeneration *in vivo* (Van Dyke et al., 2015).

### 7.6 Targeting SPM catabolism and biosynthesis: soluble epoxide hydrolase inhibition as a complementary strategy (preclinical evidence)

Rather than administering exogenous SPMs or their stable analogs, an alternative pharmacological strategy is to raise endogenous SPM output by inhibiting enzymes that limit the availability of shared lipid precursors. Soluble epoxide hydrolase (sEH) rapidly converts epoxyeicosatrienoic acids (EETs) - cytochrome-P450-derived bioactive lipid autacoids - into far less active dihydroxyeicosatrienoic acids (DHETs); because EET metabolism and SPM biosynthesis draw on overlapping polyunsaturated fatty acid pools and enzymatic machinery, sEH inhibition has been shown, in non-cancer inflammatory disease models, to increase tissue levels of lipoxin A4, resolvin E1, resolvin E2, and resolvin D6, together with the expression of their receptors (LTB4R1, ChemR23/CMKLR1, ALX/FPR2) (Abdalla et al., 2023). In a murine model of experimental periodontitis - an inflammatory condition with mechanistic parallels to unresolved tumor-associated inflammation - sEH inhibition similarly increased pro-resolving mediator levels and helped restore T-cell balance (Abdalla et al., 2024). Directly relevant to oncology, pharmacological inhibition of sEH has recently been shown to enhance the efficacy of cancer immunotherapy in preclinical models, providing an initial proof of concept that manipulating this upstream axis, rather than the SPM pathway itself, can translate into an antitumor benefit (Kelly et al., 2024). Because sEH inhibitors are already in clinical development for cardiovascular and metabolic indications (Imig and Hammock, 2009; Inceoglu et al., 2007), this pathway may offer a more pharmacologically tractable route to raising endogenous SPM tone than direct administration of intrinsically labile SPMs (see Section 7.5); head-to-head comparisons between the two strategies in oncology models are still lacking.

### 7.7 Candidate biomarkers for patient selection and response monitoring

Translating SPM biology into oncology practice will require biomarkers capable of identifying patients likely to benefit from resolution-targeted strategies and of monitoring pharmacodynamic response. Based on the direct cancer evidence reviewed in Sections 6.3, 6.4 and Table 1, several non-mutually-exclusive candidate biomarker classes emerge: (i) Tissue or plasma SPM/PIM profiles, measured by targeted LC-MS/MS, including the resolvin, protectin, and maresin species and their precursors (e.g., 15-HEPE, 18-HEPE, 20-HDOHE, 7-HDOHE, RvE2, protectin DX) that were significantly reduced by chemotherapy and correlated with survival in MPM (Gatti et al., 2026); (ii) Biosynthetic enzyme expression, particularly ALOX5/ALOX15 (governing the balance between leukotriene and lipoxin/resolvin output) and EPHX1, both of which are consistently under-expressed in tumor *versus* normal tissue in colorectal cancer and MPM (Soundararajan et al., 2025; Gatti et al., 2026); (iii) A composite, multi-gene prognostic signature, exemplified by the five-gene CPS (CYP3A4, ALOX15, EPHX1, GSTM4, GPX4) validated in the TCGA mesothelioma cohort, which stratified patients by overall survival independent of clinical covariates (Gatti et al., 2026); and (iv) Neutrophil- and macrophage-related markers, including the SAA/ALOX5-driven tumor-associated neutrophil signature described in lung cancer (Liu et al., 2016) and the M1-macrophage enrichment associated with low-CPS, high-risk MPM (Gatti et al., 2026), reflecting the immune consequences of defective resolution rather than SPM levels *per se*. None of these candidates has yet been prospectively validated as a predictive biomarker for an SPM-directed intervention, and head-to-head comparisons across tumor types are lacking; we present them here as a rational starting set for biomarker-anchored clinical trial design rather than as clinically ready assays.

### 7.8 Adjunct benefits: SPM-based mitigation of cancer- and therapy-associated pain (preclinical evidence, non-tumor-growth endpoint)

Beyond their direct and indirect antitumor actions discussed above, several resolvins have shown analgesic efficacy in preclinical models of cancer-associated pain. This distinct and clinically important endpoint is independent of tumor growth inhibition. In murine models of bone cancer pain, intrathecal administration of resolvin D1 and resolvin E1 reduced heat and mechanical hyperalgesia (Khasabova et al., 2020), and systemic resolvin D1 reduced cancer-related bone pain irrespective of sex (Flippen et al., 2024). Resolvin D2 has also been reported to reduce chemotherapy-induced neuropathic pain as well as bone cancer pain, through spinal inhibition of IL-17 secretion, reduced CXCL1 release, and dampened astrocyte activation (Pang et al., 2023). In the oral cavity, resolvin D2 exhibited both anti-tumor and analgesic effects in a model of oral squamous cell carcinoma, reducing tumor size alongside pro-inflammatory cytokine and chemokine levels (Ye et al., 2018). These findings suggest that, independent of any effect on tumor biology, SPM-based strategies may help mitigate the substantial symptom burden - including chemotherapy-induced peripheral neuropathy and bone pain - associated with cancer and its treatment. As for the antitumor applications discussed elsewhere in this review, however, these analgesic effects have not been evaluated in oncology clinical trials and should be regarded as a preclinical, hypothesis-generating adjunct rationale rather than an established therapeutic benefit.

## 8 Limitations

This review has several limitations that should inform how its conclusions are interpreted. First, it is a narrative rather than a systematic review. At the same time, we applied and disclosed an explicit search strategy (Section 1.2), we did not perform formal risk-of-bias grading of each primary study, a duplicate-reviewer screening process, or a quantitative meta-analysis, and selection of the literature therefore retains an element of expert judgment. Second, the evidence base underlying the “failed resolution” model of cancer is markedly heterogeneous in kind and quality: it spans *in vitro* biochemistry, cancer cell-line experiments, syngeneic and genetically engineered murine tumor models, human tumor transcriptomics and lipidomics, and a small number of early-phase clinical observations, and, as flagged throughout the text and summarized in Table 1, only a minority of the claims discussed here are supported by direct, cancer-specific causal evidence; much of the mechanistic framework linking SPMs to macrophage reprogramming, neutrophil dynamics, and stromal modulation (Section 4) is extrapolated from non-cancer models of inflammation and wound healing and should be regarded as biologically plausible rather than established in the tumor context. Third, clinical data on SPM-based strategies in oncology remain essentially absent: to our knowledge, no SPM-directed intervention has been tested in a completed oncology clinical trial, and the translational claims made throughout this review (Sections 6–7) should be read as a rationale for future hypothesis testing rather than as evidence of clinical efficacy or of a validated therapeutic pathway. Fourth, several of the translational conclusions we discuss - including the possibility that restoring pro-resolving signaling could mitigate chemotherapy resistance - rest mainly on preclinical, indirect, or hypothesis-driven evidence and have not been shown to reverse therapy resistance in a clinically meaningful oncology setting; we have revised the text throughout to make this distinction explicit rather than implying causal, clinically validated relationships. Finally, the biomarker panel proposed in Section 7.7 is derived from a small number of cohorts (in one case, n = 19 patients). It requires independent, prospective validation before any clinical use can be considered. Likewise, the analgesic effects of SPMs described in Section 7.8 have been demonstrated only in preclinical pain models and represent a distinct, non-oncologic endpoint that should not be conflated with antitumor efficacy.

## 9 Conclusions and future perspectives

Current evidence supports a conceptual shift from viewing cancer as a condition in which the endogenous program of resolution fails to be completed. Within this framework, the tumor microenvironment appears as a tissue compartment locked in an unresolved inflammatory state. Sustained prostaglandin- and leukotriene-driven signaling, neutrophil persistence, defective efferocytosis, aberrant angiogenesis, and fibro-inflammatory remodeling all fit (Liotti et al., 2022; Fishbein et al., 2021). This perspective suggests that SPMs have the potential to simultaneously reshape multiple compartments of the tumor microenvironment, including macrophage programming, neutrophil behavior, and endothelial and stromal responses (Lavy et al., 2021; Mattoscio et al., 2021). At the same time, these effects are unlikely to be uniform across tumors, because receptor expression and ligand responsiveness are context-dependent and may vary substantially across tissues, stages, and cellular compartments (Chiang and Serhan, 2020; Pirault and Bäck, 2018).

The tissue-specific distribution and, possibly, a tumor stage- and grade-dependent imbalance in PIM/SPM may determine the relative weight of metabolic reprogramming, immune dysfunction, and stromal permissiveness, thereby contributing to the heterogeneous landscape of tumor persistence and therapeutic resistance (Soundararajan et al., 2025; Fishbein et al., 2021). Human multi-omics data support this interpretation, showing an unbalanced PIM/SPM ratio that follows the temporal progression of the disease in mouse models (Satpathy et al., 2015). Therefore, while the central question is no longer whether resolution pathways matter in cancer, where the tractable point of control lies is the main future challenge (Lavy et al., 2021; Fishbein et al., 2021). Still, a substantial translational gap remains. Evidence is growing from tumor metabololipidomics (Wang and Dubois, 2010), circulating mediator studies (de Oliveira et al., 2024; Sun et al., 2009), and precursor-based interventional work (Hasturk et al., 2021). These signals remain early and insufficient to define a clinically validated SPM-directed strategy, due to both the described translational obstacles and a lack of knowledge. Within this respect, major questions lie the ground for investigation: for example, 1) Is it worth studying the cancer stage- and grade- specific distribution? 2) Is it possible to identify cancer-specific defective states that help the disease progress? When SPMs restrain tumor growth in preclinical settings, what is the dominant effector compartment: the spatial interplay between TME, tumor cells, and untransformed counterparts? Addressing such questions would aid in determining optimal biomarker-scouting strategies and patient-stratification modalities to translate resolution biology into actionable oncology.
