# Supplementary material for: Specialized pro-resolving mediators and cancer: news and promises from the other side of the moon
Source: Front Pharmacol. 2026 Oct 1;17:1907377. doi: 10.3389/fphar.2026.1907377 (PMC13669855; doi:10.3389/fphar.2026.1907377)
Supplement: Supplementary file 1 [file Table1.docx]

**Supplementary Table S1. Additional cancer-type-specific preclinical evidence for SPM-based strategies**

Table 2 (section 6.4) summarizes direct evidence for an unresolved PIM/SPM balance in specific tumor types. The table below complements it by summarizing additional cancer-type-specific interventional and mechanistic studies discussed in sections 4.3, 7.2, 7.3, 7.6, and 7.8, spanning tumor-growth-inhibition, immune-reprogramming, and analgesic (non-tumor-growth) endpoints; as elsewhere in this review, all entries below remain preclinical and none has been evaluated in a completed oncology clinical trial.

**Supplementary Table S1.** Additional cancer-type-specific preclinical evidence for SPM-based antitumor and analgesic strategies discussed in this review.

| **Cancer type/condition** | **Model/sample** | **SPM(s) studied** | **Intervention or measurement** | **Key finding** | **Evidence tier** | **Ref.** |
| --- | --- | --- | --- | --- | --- | --- |
| **Cervical cancer (HPV-positive)** | Human & murine HPV-positive cervical cancer; in vitro and in vivo, with pharmacological neutrophil depletion | RvD1 | RvD1 administration; TCGA immune-infiltration deconvolution | Reduced tumor growth via neutrophil-dependent recruitment of anti-tumor monocytes; higher intratumoral monocyte infiltration linked to better clinical outcome | Direct (in vivo/in vitro) + indirect (TCGA correlation) | (48) |
| **Prostate cancer** | Pten-null genetically engineered mice; dietary DHA vs. ω-6-enriched diet | RvD1, RvD2 | Dietary modulation; TAM polarization marker analysis (CD206, VEGFA, EGF) | Slowed disease progression; effect attributed to macrophage reprogramming rather than a direct tumor cell-autonomous mechanism | Direct (murine) | (46) |
| **Hepatocellular carcinoma** | Human HCC cell lines co-cultured with cancer-associated fibroblasts (CAFs) | RvD1 | Inhibition of CAF-derived paracrine COMP secretion | Reduced epithelial-mesenchymal transition and stem-like features of HCC cells | Direct (in vitro, human cells) | (55) |
| **Non-small cell lung cancer (KRAS-driven)** | Murine KRAS-driven lung adenocarcinoma model, combined with cisplatin | RvE1 | RvE1 + cisplatin combination | Enhanced tumor growth inhibition vs. either agent alone; reduced COX-2/NF-κB/VEGF-A/Ang-1/Ang-2, reduced TAM/TAN infiltration, lower neutrophil-to-lymphocyte ratio | Direct (murine, combination therapy) | (60) |
| **Bone cancer pain** | Murine bone cancer pain model; intrathecal administration | RvD1, RvE1 | Intrathecal dosing | Reduced heat and mechanical hyperalgesia | Direct (murine) | (126) |
| **Bone cancer pain (systemic)** | Murine bone cancer pain model; systemic administration | RvD1 | Systemic dosing | Reduced cancer-related bone pain, independent of sex | Direct (murine) | (127) |
| **Chemotherapy-induced neuropathic pain and bone cancer pain** | Murine pain models | RvD2 | Spinal/systemic administration | Reduced pain via inhibition of spinal IL-17 secretion, CXCL1 release, and astrocyte activation | Direct (murine) | (128) |
| **Oral squamous cell carcinoma** | Murine OSCC model | RvD2 | Combined tumor-growth and pain endpoints | Reduced tumor size and pro-inflammatory cytokines/chemokines, with a concurrent analgesic effect | Direct (murine) | (73) |
| **Pan-cancer (immunotherapy-enhancing strategy)** | Preclinical tumor immunotherapy models | Endogenous SPMs, raised via soluble epoxide hydrolase (sEH) inhibition | Pharmacological sEH inhibition | Enhanced efficacy of cancer immunotherapy | Direct (murine, mechanistic) | (121–123) |

As with Table 2, no entry in this table constitutes evidence of clinical efficacy; all studies are preclinical (cell-based or murine) unless otherwise noted, and are presented to summarize the biological rationale for the corresponding SPM-based strategies.
